# A new approach to primer design for the control of PCR bias in methylation studies

**DOI:** 10.1186/1756-0500-1-54

**Published:** 2008-07-28

**Authors:** Tomasz K Wojdacz, Lise Lotte Hansen, Alexander Dobrovic

**Affiliations:** 1 Molecular Pathology Research and Development Laboratory, Department of Pathology, Peter MacCallum Cancer Centre, St. Andrews Place, East Melbourne, Vic 3002, Australia; 2 Institute of Human Genetics University of Aarhus The Bartholin Building, DK-8000, Aarhus C, Denmark; 3 Department of Pathology, University of Melbourne, Parkville, Vic 3010, Australia

## Abstract

Primer design for PCR-based methylation analysis following bisulfite conversion of DNA is considerably more complex than primer design for regular PCR. The choice of the optimal primer set is critical to the performance and correct interpretation of the results. Most methodologies in methylation analysis utilize primers that theoretically amplify methylated and unmethylated templates at the same time. The proportional amplification of all templates is critical but difficult to achieve due to PCR bias favouring the amplification of the unmethylated template. The focus of this brief communication is to point out the important criteria needed for the successful choice of primers that will enable the control of PCR bias in bisulfite based methylation-screening protocols.

## Findings

With the increased awareness of the central role of epigenetic mechanisms in development and cancer, many techniques for the analysis of DNA methylation have been developed [1,2]. The overwhelming majority of these techniques involve sodium bisulfite modification of the DNA template, followed by PCR amplification of the region of interest.

The primers for analysis of DNA methylation status either seek (i) to interrogate the methylation of the CpG sites within the primer binding site e.g. methylation specific PCR (MSP) primers or, (ii) to amplify the region of interest regardless of its methylation status allowing for post-PCR determination of the methylation of the region of interest e.g. methylation independent PCR (MIP) primers.

In MSP, assessment of the methylation status of a given locus is determined by the CpG sites within the primer sequence. It is thus important to include several CpG sites towards the 3' end of the primers to ensure specific binding and subsequent amplification of only methylated variants of the template. Careful evaluation of MSP primers has to be performed prior to analyses to assure the specificity and exclude over interpretation of results.

MIP primers are required for applications in which the determination of methylation status of the sequence of interest is performed after the PCR. The post-PCR determination of the methylation status of amplified sequences can be performed in various ways such as: sequencing [3,4], restriction digestion [5,6], DHPLC [7], single strand conformation analysis [8,9], melting curve analysis [10,11] or high resolution melting [12,13].

The design of MIP primers that will proportionally amplify methylated and unmethylated template is the main challenge for these analyses. In practice, true methylation independence is often not achievable due to PCR bias, which favours the amplification of unmethylated templates.

The PCR bias phenomenon in which methylated and unmethylated sequences are not proportionately amplified was first bought to general attention by Warnecke et al [14]. Various technical modifications were tried to limit PCR bias but only the use of the Stoffel fragment of Taq polymerase gave a limited degree of success [14]. Subsequently, the issue of bias was largely ignored in the methylation literature. Nevertheless it is important to ensure that methylated and unmethylated sequences are proportionately amplified in order to have confidence that the results reflect biological reality.

Recommendations for primer design have been made to enable unbiased amplification from both methylated and unmethylated alleles in a proportional fashion [4] and these have been adopted by most laboratories. It was recommended not to include any CpG dinucleotides into the primer sequence so that the primers would bind equally to both methylated and unmethylated templates. If it were impossible to avoid CpGs in primer sequences, it was recommended that the Cs of CpG residues should be replaced by a mismatched base corresponding to either C (methylated sequences) or T (unmethylated sequences).

Recently, it has been confirmed that PCR bias is a major problem in methylation analysis and that strong bias towards unmethylated sequences occurs in most PCR amplifications that use methylation independent primers designed according to guidelines which exclude CpG residues from the primers [10,15].

The optimisation of annealing temperature of PCR amplification has been shown to improve the amplification of methylated template in an unmethylated background [15]. Nevertheless in our experience (as in the previous report by Warnecke et al. [14]), optimisation of the annealing temperature did not in most cases improve the amplification of methylated template [10].

We have chosen a different strategy to improve the amplification of methylated templates and have shown that inclusion of limited CpG residues into the primer sequences and optimisation of annealing temperature of PCR amplification can correct for the bias that occurs during PCR amplification of the sequences [10,12]. The degree of bias correction can be manipulated by varying the annealing temperature to control the stringency of binding of the primers to the template.

At lower annealing temperatures, there is little favouring of the methylated template by the primers. At higher annealing temperatures, the primers will bind almost exclusively to the methylated template allowing the reversal of PCR bias and amplifying mostly methylated template. At an intermediate annealing temperature, the primer annealing bias that favours methylated templates will compensate for the amplification bias that favours unmethylated templates [12].

The design of the primers that enable the above described correction for PCR bias is different from the one used in currently available freeware programs for primer design in methylation experiments. Those programs thus are not suitable for use in design of the primers with limited numbers of CG sites as they either design primers specific to the methylated template for MSP experiments or primers without CGs for MIP experiments.

The primers that enable correction for PCR bias should be designed as follows:

1. Include a limited number of CpG dinucleotides (usually one) in the primer sequence. In our experience, up to two or rarely even three CpGs can be included in each primer before the primers become entirely selective for methylated templates and thus amplify only the methylated sequence. The ability to include limited CpGs can make MIP primers easier to design on the CpG rich sequences that are the usual targets for methylation analysis.

2. The included CpGs should as far as possible from the 3' end of the primers as otherwise the primers will be entirely selective for methylated templates and amplify only methylated sequence. In most cases this has to be determined empirically.

3. The salt adjusted melting temperature (T_m_) of the primer should be around 65°C in order to run the PCR at or near to 60°C. Running the PCR in this temperature range is important to ensure the specificity of the PCR reaction. The T_m _of primers can be calculated using Oligonucleotide Properties Calculator . The salt adjusted T_m _has been the most accurate one in our experience. Primer Express (Applied Biosystems, Foster City, CA) also works well. Ideally the primer T_m_s should be matched within 1°C.

4. The inclusion of one or more Ts originating from a non-CpG C at, or near the 3' end of each primer is desirable to ensure amplification of only bisulfite modified DNA.

5. The selected primers should be further evaluated in regard to standard parameters for primer design e.g. secondary structure, primer dimer formation. To evaluate those primer features, tools for primer design like Amplify  can be used.

To test the primer set for the extent of the bias at various annealing temperatures, we PCR amplify a range of dilutions of a fully methylated DNA template into unmethylated DNA. The proportion of methylated to unmethylated sequences in the PCR product can then be estimated by melting analysis and the choice of annealing temperature which allows correction for PCR bias can be empirically determined [10,12]. In addition, the primer set has to be tested with non-bisulphite treated DNA as a template to eliminate the possibility that it amplifies PCR products from unconverted DNA.

It has been generally recognised that MSP can give higher positivity for methylation than MIP based methods. This has been attributed to the higher sensitivity and tendency to false positives of MSP. What has been less generally recognised is that MIP based techniques using MIP primers designed according to the commonly followed guidelines may fail to detect methylation at biologically significant levels [10]. The discrepant results obtained between the two approaches has been reported [16].

In conclusion, primer design for methylation studies is a complex task for MIP based protocols. Careful design and subsequent optimisation of the primer set has to be performed and each primer set has to be treated individually. Optimisation has to address both the PCR cycling conditions and the components of the PCR reaction to choose the optimal protocol for high performance for a given primer pair. The PCR bias in MIP based experiments in our experience was the main problem compromising these analyses. The guidelines for primer design presented here should assist in the design of methylation detection experiments whenever MIP primers are used such as bisulfite sequencing and nearly all methylation screening protocols including MS-HRM.

## Competing interests

The authors are co inventors on patent applications on aspects of the MS-HRM methodology. The patents have been filed for by University of Aarhus and Peter MacCallum Cancer Centre.

## Authors' contributions

TKW performed the experiments lending to this publication under the supervision of LLH and AD. All authors were involved in writing of this manuscript.
